# Ovulatory Recovery following weight loss in women with polycystic ovary syndrome and obesity: a *post hoc* analysis of the BAMBINI randomised controlled trial

**DOI:** 10.1093/humrep/deag037

**Published:** 2026-03-11

**Authors:** Suhaniya N S Samarasinghe, Ali Abbara, Patricia M Ortega, Ahmed R Ahmed, Christos Tsironis, Sanjay Purkayastha, Vinod Menon, Harpal Randeva, Alexander D Miras

**Affiliations:** MRC Laboratory of Medical Sciences (LMS), Imperial College London, London, UK; Department of Metabolism, Digestion and Reproduction, Imperial College London, London, UK; Department of Metabolism, Digestion and Reproduction, Imperial College London, London, UK; Imperial Weight Centre, Imperial College Healthcare NHS Trust, London, UK; Department of Surgery, Imperial College London, London, UK; Department of Surgery, Imperial College London, London, UK; Imperial Weight Centre, Imperial College Healthcare NHS Trust, London, UK; Division of Life Sciences, Brunel University, London, UK; Department of Surgery, University Hospital Coventry and Warwickshire NHS Trust, Coventry, UK; Warwick Medical School, University of Warwick, Warwick, UK; Warwick Medical School, University of Warwick, Warwick, UK; Warwickshire Institute for the Study of Diabetes, Endocrinology and Metabolism, University Hospitals Coventry and Warwickshire NHS Trust, Coventry, UK; School of Medicine, Ulster University, Derry, UK

**Keywords:** PCOS, ovulation, obesity, weight loss, surgery, lifestyle

## Abstract

**STUDY QUESTION:**

What is the frequency of ovulatory recovery (OvR) after different degrees of total weight loss (TWL) in women with polycystic ovary syndrome (PCOS) and obesity, and can an excessive degree of TWL be identified that is harmful to the chance of OvR?

**SUMMARY ANSWER:**

Any degree of TWL was associated with a higher likelihood of OvR, and no upper threshold of TWL associated with reduced OvR was identified.

**WHAT IS KNOWN ALREADY:**

Modest weight loss (5–10%) improves reproductive function in women with PCOS. However, the relationship between greater degrees of TWL and OvR remains uncertain.

**STUDY DESIGN, SIZE, DURATION:**

Secondary *post hoc* analysis of a multicentre, open-label, randomised controlled trial (BAMBINI) conducted in the UK between February 2020 and April 2023. Eighty women were randomised (1:1) to standard medical care or vertical sleeve gastrectomy. Seventy-five were included in this analysis and followed up for 52 weeks.

**PARTICIPANTS/MATERIALS, SETTING, METHODS:**

Participants had PCOS, a BMI of 35 kg/m^2^ or higher, and oligomenorrhea/amenorrhoea. OvR was defined as two consecutive biochemically confirmed ovulatory events (serum progesterone 16.0 nmol/l or higher), occurring 3–5 weeks apart within the 52 week follow up period. Associations between TWL, reproductive hormones, and OvR were analysed using logistic regression. Analyses were exploratory and not prespecified.

**MAIN RESULTS AND THE ROLE OF CHANCE:**

At 52 weeks, 50.8% (38/75) achieved OvR. OvR occurred in 19% of participants without weight loss and in >50% of those who lost weight. Each 1% reduction in body weight was associated with a 5.6% increase in the odds of OvR (OR 0.944, 95% CI 0.900–0.990). Higher baseline serum anti-Müllerian hormone (OR 0.963, 95% CI [0.938–0.988]; *P* = 0.004) and higher plasma total testosterone (OR 0.324, 95% CI [0.142–0.742]; *P* = 0.008) were associated with lower odds of OvR. Greater TWL following bariatric surgery was associated with increased sex hormone-binding globulin and reduced free androgen index.

**LIMITATIONS, REASONS FOR CAUTION:**

This was an exploratory *post hoc* analysis not designed to define optimal or upper TWL thresholds. The study was not powered to detect potential adverse reproductive effects at higher degrees of TWL.

**WIDER IMPLICATIONS OF THE FINDINGS:**

These findings suggest that OvR in women with PCOS and obesity improves progressively with increasing TWL, supporting weight loss strategies including bariatric surgery in appropriately selected women not seeking imminent pregnancy.

**STUDY FUNDING/COMPETING INTEREST(S):**

The Jon Moulton Charity Trust funded the BAMBINI trial. This work was supported by grants from the National Institute of Health Research (NIHR), the NIHR/Wellcome Trust Imperial Clinical Research Facility, and the NIHR Imperial Biomedical Research Centre. The Section of Endocrinology and Investigative Medicine was funded by grants from the Medical Research Council (MRC), Biotechnology and Biological Sciences Research Council (BBSRC), and the NIHR, and was supported by the NIHR Biomedical Research Centre Funding Scheme. The views expressed are those of the author(s) and not necessarily those of the MRC, the NHS, the NIHR, or the Department of Health. S.N.S.S. was supported by an Imperial post-doctoral post-CCT Fellowship. A.A. was supported by an NIHR Clinician Scientist Award CS-2018-18-ST2-002. All authors acknowledge infrastructure support for this research from the NIHR Imperial Biomedical Research Centre (BRC).

A.D.M. has received research funding from the Medical Research Council (MRC), National Institute for Health and Care Research (NIHR), Jon Moulton Charitable Foundation, PEACEPLUS programme (EU and UK government), Anabio, Fractyl, Boehringer Ingelheim, Eli Lilly, Gila, Randox, and Novo Nordisk. A.D.M. has received honoraria for lectures and presentations from Novo Nordisk, AstraZeneca, Currax Pharmaceuticals, Boehringer Ingelheim, Screen Health, GI Dynamics, Algorithm, Eli Lilly, Ethicon, and Medtronic. A.D.M. is a shareholder in the Beyond BMI clinic, which provides clinical obesity care. H.R. is on the advisory board for Novo Nordisk and is the national lead for the REDEFINE 3 trial.

**TRIAL REGISTRATION NUMBER:**

N/A.

## Introduction

Polycystic ovary syndrome (PCOS) is the commonest endocrinopathy in women of reproductive age, estimated to affect between 11% and 13% of women worldwide (Gao *et al.*, 2025), with a large proportion of affected women remaining undiagnosed. PCOS is the leading cause of anovulatory infertility, accounting for up to 90% of cases (Balen and Rutherford, 2007).

Although obesity does not form part of the diagnostic criteria, it exacerbates the metabolic, reproductive, and psychological features of PCOS (Teede *et al.*, 2018). Obesity further exacerbates insulin resistance, hyperandrogenism, the risk of anovulatory infertility (Rich-Edwards *et al.*, 2002), and lengthens the time to conception (Gesink Law *et al.*, 2007). The current International Guidelines for the Diagnosis and Management of Polycystic Ovary Syndrome 2023 recommend lifestyle modification as the initial management of overweight/obesity, and states that bariatric/metabolic surgery could be considered to improve ‘irregular menstrual cycles, ovulation, and pregnancy rates in women with PCOS’ (Teede *et al.*, 2023).

Based on data from small studies, modest weight loss of as little as 5% has been suggested to improve metabolic outcomes and rates of ovulation (Kiddy *et al.*, 1992; Crosignani *et al.*, 2003). However, most of these studies relied on dietary interventions that rarely achieved or sustained greater weight loss. A theoretical concern is that excessive weight loss could induce a state of relative energy deficiency, which may impair reproductive function and exacerbate anovulation. This is supported by evidence that when functional hypothalamic amenorrhea (FHA) and PCOS coexist, the FHA phenotype tends to predominate (Phylactou *et al.*, 2021).

The BAMBINI randomized clinical trial published in the Lancet in 2024 was the first to randomize 80 women with anovulatory PCOS and obesity (BMI ≥35 kg/m^2^) to either best medical therapy (e.g. metformin ±orlistat but prior to widespread availability of GLP-1 receptor agonists) or bariatric surgery (sleeve gastrectomy) (Samarasinghe *et al.*, 2024a). Women in the surgical group experienced 28.5% weight loss, whereas those in the medical group remained weight-stable at 52 weeks after randomization (Samarasinghe *et al.*, 2024a). The primary outcome was the number of biochemically determined ovulatory events during weekly biochemical monitoring over 1 year, not timed to the menstrual cycle. Bariatric surgery increased the number of spontaneous ovulations by 2.5-fold compared with the medical group (incidence rate ratio 2.5 [95% CI 1.5–4.2], *P* < 0.0007) (Samarasinghe *et al.*, 2024a). Given the wide range of weight changes observed in the BAMBINI trial, including greater degrees of weight loss than those reported in previous studies, these data provided an opportunity to explore the relationship between the magnitude of weight loss and recovery of ovulatory function. More specifically, they enable exploratory assessment of whether OvR varies with the degree of weight loss and whether more substantial weight loss is associated with different likelihoods of recovery.

Here, we performed a *post hoc* secondary analysis of the BAMBINI trial to examine associations between percentage total weight loss (TWL) and OvR. Additionally, we explored whether baseline characteristics, including the degree of obesity, and biochemical features of PCOS, including anti-Müllerian hormone (AMH) or the androgen profile, or if the response to treatment (e.g. change in weight/hormonal profile) predicted the chance of OvR. As a secondary analysis that was not prespecified, these data should be regarded as hypothesis-generating to inform future larger prospective studies.

## Materials and methods

### Study design

**Figure 1.**
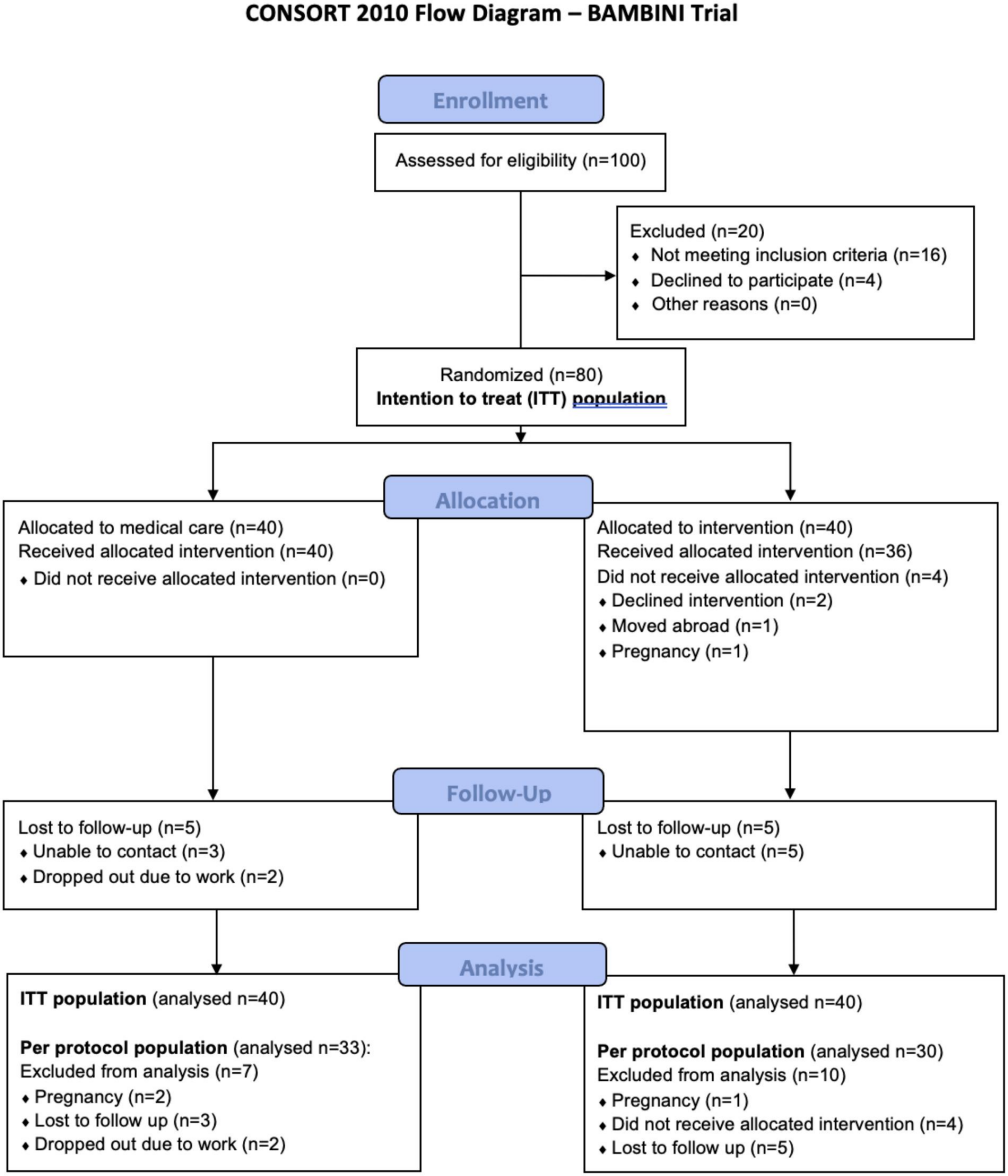
CONSORT 2010 flow diagram for the BAMBINI trial (Samarasinghe *et al*., 2024a).

The original study, the BAMBINI clinical trial (Samarasinghe *et al.*, 2024a), was an open-label randomised controlled trial (RCT) conducted in two academic clinical centres in the UK between 20 February 2020 and 17 April 2023. The trial protocol was prospectively registered (https://www.isrctn.com/ISRCTN16668711) and had approval by the London-Dulwich Research Ethics Committee (reference number 19/LO/1540). Eighty women ≥ 18 years of age with a diagnosis of PCOS based on the 2018 international evidence-based guidelines for assessment and management of PCOS (Teede *et al.*, 2018), a BMI ≥ 35 kg/m^2^, and oligomenorrhoea or amenorrhoea were recruited. Key exclusion criteria were a diagnosis of diabetes, the inability to maintain effective non-hormonal contraception, pregnancy and breastfeeding, gastro-oesophageal disease. The full exclusion criteria is in the protocol published online. Participants were randomly assigned using a computer-generated randomisation sequence in a 1:1 ratio to either medical care or surgery (Fig. 1). Medical care consisted of lifestyle modification provided by a specialist dietician, and participants were offered oral pharmacotherapy, including metformin, orlistat, or both. Surgery was a laparoscopic sleeve gastrectomy performed using a standardised technique.

This was an open-label study, and the primary endpoint for the original study was defined as a rise in serum progesterone to 16 nmol/l or more, indicative of ovulation (Leiva *et al.*, 2015). Progesterone sampling was not timed with the menstrual cycle.

This is a secondary, *post hoc* analysis to explore the association between the degree of TWL and ovulatory recovery (OvR), and to identify potential baseline and treatment-related factors associated with OvR. For the purpose of this exploratory analysis, OvR was defined as two or more consecutive biochemically confirmed ovulatory events (progesterone ≥16.0 nmol/l) occurring 3–5 weeks apart during the 52-week follow-up period.

### Statistical analysis

Of 80 women randomised in the original trial, 75 were included in this secondary *post hoc* analysis (4 did not receive their intervention (surgery) and 1 withdrew due to conception within 1 month of the intervention starting [medical care]). Associations between OvR and weight gain or % TWL categories (0–5, 5–10, 10–15, 15–20, ≥ 20) were examined using Firth’s penalised likelihood logistic regression, with the reference group being those who gained/did not lose weight. %TWL was compared between ovulatory and anovulatory patients within both medical care and surgery groups at 3, 6, and 12 months. We used logistic regression (separate analysis models) to evaluate the odds of OvR using predictors such as baseline weight and relevant reproductive hormone concentrations prior to randomisation. Multiple analyses may increase the risk of type 1 error in this small population. We also analysed whether variables reflecting the response to treatment predicted OvR, including %TWL and change in hormone concentrations at the point of OvR (for the ovulatory group) and 12 months (for the anovulatory group). As this is a secondary *post hoc* analysis, *P*-values are nominal and should be interpreted with caution.

Hormone levels were measured monthly. Changes in hormone concentrations over 476 reported menstrual cycles were analysed using a mixed-effects model. All statistical analyses were conducted using Stata software, version 17 BE (StataCorp).

### Role of the funding source

The study funder had no role in study design, data collection, data analysis, data interpretation, or writing of the paper.

## Results

Seventy-five participants (39 in the medical group and 36 in the surgical group) from the BAMBINI (Samarasinghe *et al.*, 2024a) RCT were included in this secondary *post hoc* analysis, of whom 38 (50.7%) had OvR within 52 weeks (Table 1).

**Table 1.** Number of participants in each treatment group versus ovulatory status.

Treatment	Anovulatory	Ovulatory	Total
Medical care	27 (69.2%)	12 (30.8%)	39
Surgery	10 (27.8%)	26 (72.2%)	36
**Total**	37	38	75

### Weight loss and OvR

Across the cohort, OvR occurred more frequently among participants who achieved at least some weight loss, with the greatest proportion observed among those with moderate weight loss of 10–15%, independent of baseline BMI (Table 2). Independent of %TWL, surgery was associated with higher odds of OvR compared to medical care (OR 5.850, 95% CI 2.158–15.857; *P* = 0.001).

**Table 2.** Percentage change in weight (TWL) and regain of ovulatory status (OvR).

Group	Total	Anovulatory	Ovulatory	Odds ratio (OR) versus Group 0	95% CI	*P*-value
**Group 0 (Weight increase)**	21	17 (81%)	4 (19%)	–	–	–
**TWL Group 1 (0–5 %)**	14	6 (43%)	8 (57%)	5.085	1.187, 21.782	0.028
**TWL Group 2 (5–10%)**	8	4 (50%)	4 (50%)	3.889	0.734, 20.606	0.110
**TWL Group 3 (10–15%)**	12	0	12 (100%)	97.222	4.790, 1973.47	0.003
**TWL Group 4 (15–20%)**	8	4 (50%)	4 (50%)	3.889	0.734, 20.606	0.110
**TWL Group 5 (≥20%)**	12	5 (42%)	7 (58%)	5.303	1.170, 24.035	0.030

Firth logistic regression was used to compare OvR in comparison to group 0 (gained weight following randomization).

**Figure 2.**
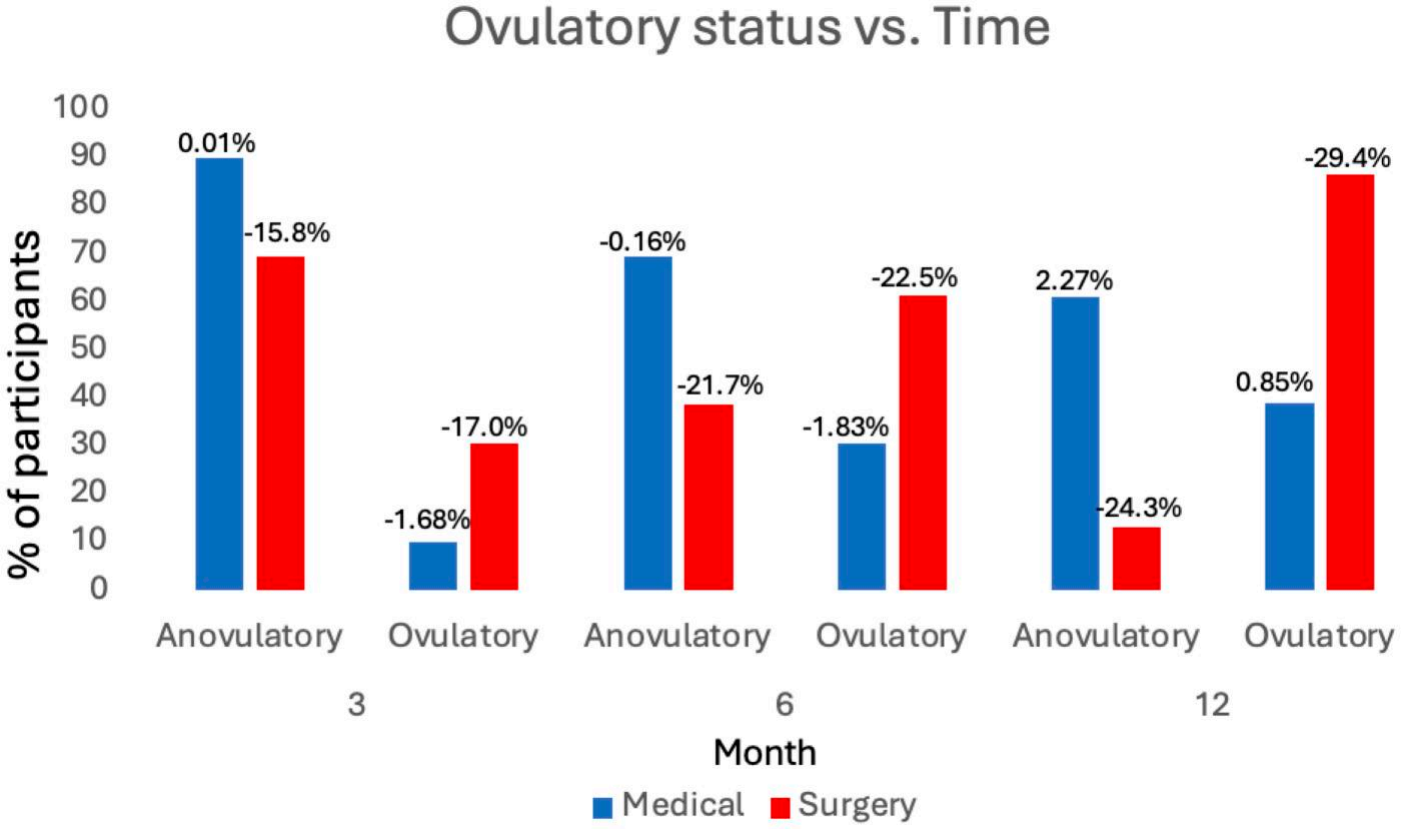
**Ovulatory status versus time in months (3, 6, and 12 months) for medical care (blue) and surgery (red).** The percentage of women with ovulatory recovery (OvR) in the medical and surgical is presented at 3, 6, and 12 months after randomization. The % figures above the bar represent the mean change in weight from baseline at that time-point.

In the surgical group, weight loss increased progressively over 12 months, whereas in the medical group, weight loss was minimal (<2%) and confined to the first 6 months (Fig. 2). Within the medical group, mean percentage weight loss did not differ significantly between participants who recovered ovulation and those who remained anovulatory. At 6 months, the mean percentage weight change was −1.83% in those who regained ovulation compared with −0.16% in those who remained anovulatory (*P* = 0.35). At 12 months, the corresponding values were +0.85% and +2.27%, respectively (*P* = 0.49) (Fig. 2). In the surgical group, greater degrees of weight loss were consistently associated with higher proportions of OvR across follow-up timepoints.

#### Weight gain, %TWL, and regain of ovulation

Overall, a greater percentage of TWL was associated with higher odds of OvR (*P* = 0.034). Participants who experienced any degree of weight loss had higher odds of ROS than those who gained weight. However, when TWL was examined across the categorical subgroups, there was no statistically significant evidence of differences in OvR between TWL groups 1–5. Although the 10–15% TWL subgroup appeared to have a higher proportion of OvR, this group was small (n = 12), resulting in wide CIs and imprecise estimates. Similarly, few participants achieved ≥20% TWL, making it difficult to draw conclusions regarding an optimal TWL range (Table 2).

### Baseline predictors of OvR

**Figure 3.**
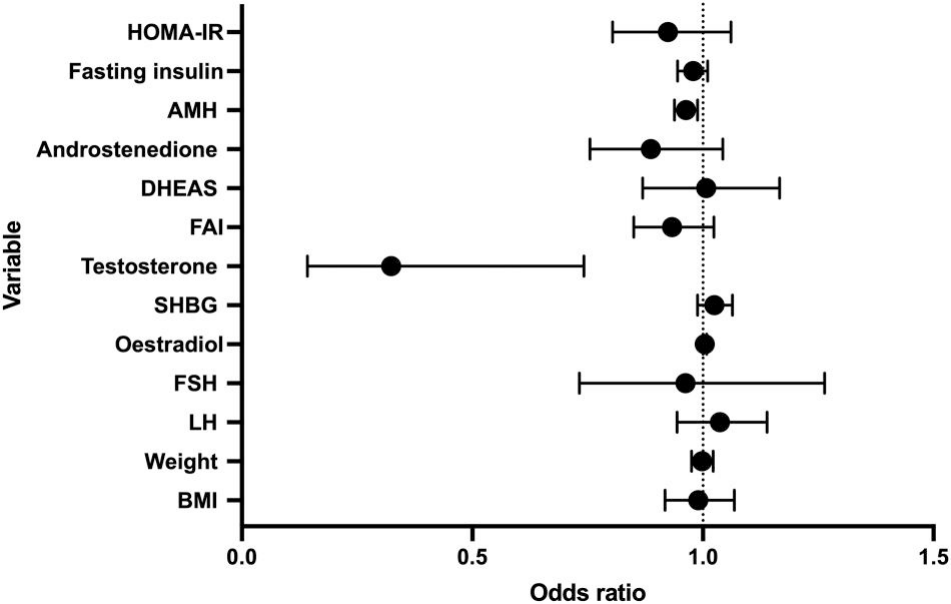
**Forest plot showing the association between baseline clinical and biochemical variables and ovulatory recovery (OvR).** SHBG, sex hormone-binding globulin; FAI, free androgen index; DHEAS, dehydroepiandrosterone sulphate; AMH, anti-Müllerian hormone; HOMA-IR, homeostatic model assessment of insulin resistance. Odds ratios (OR) and 95% CIs were derived from logistic regression models. An OR > 1 indicates higher odds of the outcome with increasing variable value, OR < 1 indicates lower odds. Vertical dotted line represents the null value (OR = 1). Variables with CIs crossing 1 were not statistically significant.

Using logistic regression analysis, higher baseline total testosterone and AMH concentrations were associated with lower odds of OvR. Each 1 nmol/l increase in total testosterone was associated with a 67.6% reduction in the odds of OvR (OR 0.324, 95% CI 0.142–0.742; nominal *P* = 0.008), and each 1 pmol/l increase in AMH was associated with a 3.7% decrease in the odds of regaining ovulation (OR 0.963, 95% CI 0.938–0.988; nominal *P* = 0.004). Higher baseline oestradiol (not timed with menstruation) was weakly associated with higher odds of OvR (OR 1.004, 95% CI 1.000–1.008; nominal *P* = 1.004). Baseline weight and BMI were not associated with ROS, and higher baseline weight did not preclude OvR following weight loss (Table 3, Fig. 3).

**Table 3.** Baseline predictors of ovulatory recovery (OvR).

Variable	Odds ratio (OR)	*P*-value	95% CI
**BMI** kg/m^2^	0.990	0.800	0.918–1.068
**Weight** kg	0.998	0.878	0.975–1.022
**LH IU/l**	1.037	0.452	0.944–1.139
**FSH IU/l**	0.962	0.780	0.732–1.264
**Oestradiol** pmol/l	1.004	0.041	1.000–1.008
**SHBG** nmol/l	1.025	0.190	0.988–1.064
**Testosterone** nmol/l	0.324	0.008	0.142–0.742
**FAI**	0.933	0.144	0.850–1.024
**DHEAS** μmol/l	1.007	0.928	0.869–1.166
**Androstenedione** nmol/l	0.887	0.148	0.755–1.043
**AMH** pmol/l	0.963	0.004	0.938–0.988
**Fasting insulin** mIU/l	0.979	0.224	0.945–1.010
**HOMA-IR**	0.924	0.266	0.804–1.061

SHBG, sex hormone-binding globulin; FAI, free androgen index; DHEAS, dehydroepiandrosterone sulphate; AMH, anti-Müllerian hormone; HOMA-IR, homeostatic model assessment of insulin resistance.

#### Does the response to weight loss management affect the chance of OvR?

**Figure 4.**
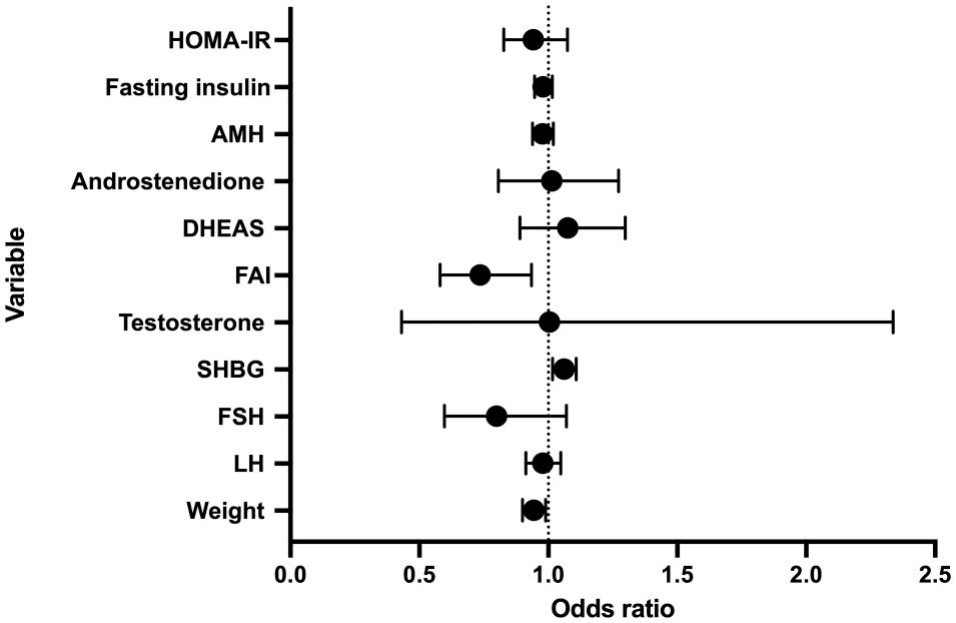
**Forest plot showing the association between change in baseline clinical and biochemical variables and ovulatory recovery (OvR).** SHBG, sex hormone-binding globulin; FAI, free androgen index; DHEAS, dehydroepiandrosterone sulphate; AMH, anti-Müllerian hormone; HOMA-IR, homeostatic model assessment of insulin resistance. Odds ratios (OR) and 95% CIs were derived from logistic regression models. An OR > 1 indicates higher odds of the outcome with increasing variable value, OR < 1 indicates lower odds. Vertical dotted line represents the null value (OR = 1). Variables with CIs crossing 1 were not statistically significant.

Exploratory logistic regression analyses examined whether treatment-related changes in weight and reproductive hormones were associated with OvR. Greater %TWL, increase in SHBG, and reduction in FAI were associated with higher odds of OvR. Each 1% reduction in body weight was associated with a 5.6% increase in the odds of OvR (OR 0.944, 95% CI 0.900–0.990; nominal *P* = 0.017). Similarly, each 1 nmol increase in SHBG was associated with a 6.1% increase in the odds of OvR (OR 1.061, 95% CI 1.016–1.108; nominal *P* = 0.007), and each 1-unit reduction in FAI was associated with a 26% increase in the odds (OR 0.736, 95% CI 0.580–0.935; nominal *P* = 0.012) (Table 4, Fig. 4).

**Table 4.** Change in clinical and biochemical variables and ovulatory recovery (OvR).

Variable	Odds ratio (OR)	*P*-value	95% CI
**Weight** kg	0.944	0.017	0.900–0.990
**LH** IU/l	0.979	0.542	0.913–1.048
**FSH** IU/l	0.799	0.133	0.597–1.070
**SHBG** nmol/l	1.061	0.007	1.016–1.108
**Testosterone** nmol/l	1.004	0.993	0.431–2.337
**FAI**	0.736	0.012	0.580–0.935
**DHEAS** μmol/l	1.075	0.453	0.890–1.298
**Androstenedione** nmol/l	1.013	0.914	0.806–1.272
**AMH** pmol/l	0.978	0.301	0.939–1.019
**Fasting insulin** mIU/l	0.980	0.259	0.947–1.015
**HOMA-IR**	0.942	0.372	0.827–1.074

SHBG, sex hormone-binding globulin; FAI, free androgen index; DHEAS, dehydroepiandrosterone sulphate; AMH, anti-Müllerian hormone; HOMA-IR, homeostatic model assessment of insulin resistance.

### Analysis of hormone concentrations during the menstrual cycle

**Figure 5.**
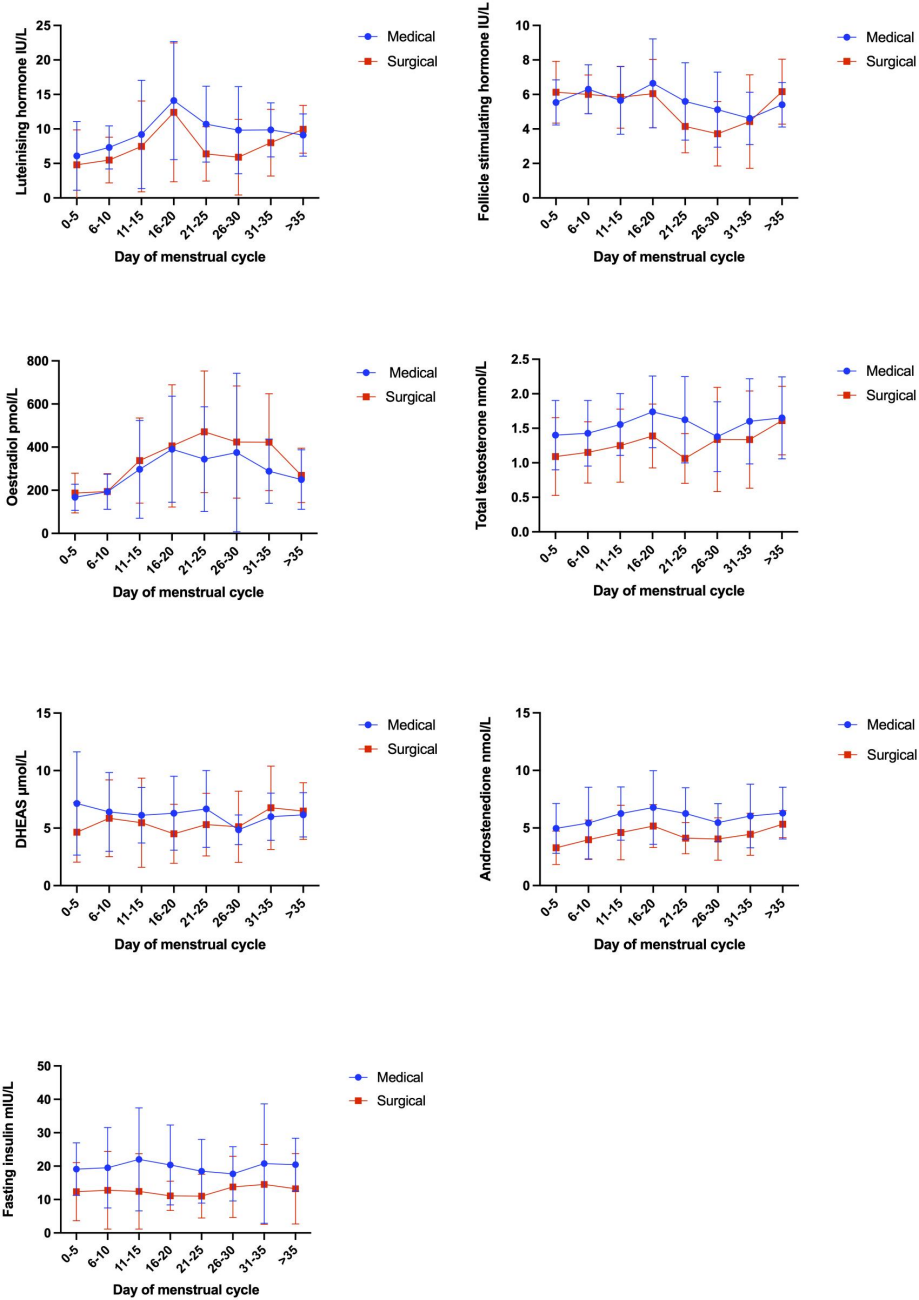
Mean hormone concentrations for the two treatment groups during the menstrual cycle.

Hormone concentrations were analysed across 476 reported menstrual cycles (206 in the medical care group and 270 in the surgery group) over a 52-week follow-up period using mixed-effects models. Some participants contributed multiple cycles during this time period. LH (Mean difference [MD] −4.319, 95% CI [−8.019 to −0.619]), total testosterone (MD −0.562, 95% CI [−0.955 to −0.168]), androstenedione (MD −2.135, 95% CI [−3.559 to −0.711]), and insulin (MD −7.434, 95% CI [−13.77 to −1.101]) concentrations were lower during the luteal phase (Days 21–25) in women who had undergone surgery than in the medical group. During Days 16–20 of the luteal phase, total testosterone (MD −0.351, 95% CI [−0.687 to −0.0159]) and fasting insulin (MD −9.222, 95% CI [−15.43 to −3.009]) concentrations were lower in the surgical group (Fig. 5).

## Discussion

In this secondary, *post hoc* analysis of the BAMBINI (Samarasinghe *et al.*, 2024a) RCT, which compared the safety and efficacy of bariatric surgery with standard medical care in women with PCOS, obesity, and oligo/amenorrhoea, we explored the association between the magnitude of weight loss, hormonal changes, and OvR. These *post hoc* analyses provide insights into the potential effects of varying degrees of weight loss and hormonal levels on OvR.

Weight loss was associated with a higher likelihood of OvR; however, the study was not powered to detect differences between specific degrees of TWL. Our findings do not define an optimal weight loss target but raise the hypothesis that even small to moderate degrees of weight loss may be sufficient for OvR in many women with PCOS and obesity. This is consistent with previous studies demonstrating reproductive and metabolic benefits 5–10% weight loss (Dzienny and Seifer, 2025).

Despite minimal overall weight loss in the medical care group, there was a significant increase in the frequency of self-reported menses from baseline to 52 weeks (median, 3–6 cycles: *P* < 0.001), which could be due to improvements in insulin resistance with metformin. The exact mechanisms underlying the therapeutic action of metformin are complex and not fully understood but in addition to its intrahepatic blood glucose lowering effects, there is strong evidence that extrahepatic sites such as the gut and its microbiota are also involved in its various clinical benefits (Foretz *et al.*, 2023). A single-cell profiling study of the human endometrium in PCOS reported extensive recovery of disease-specific endometrial signatures following 16 weeks of treatment with metformin and lifestyle management (Eriksson *et al.*, 2025).

Importantly, while all participants who achieved 10–15% TWL recovered ovulation, this observation was based on a very small subgroup and should therefore not be used to define clinical thresholds. The absence of a demonstrable adverse effect of higher degrees of weight loss on OvR is reassuring but should not be interpreted as confirmation of safety in participants achieving ≥20% TWL as the number of patients in this group was small.

Our findings are broadly in keeping with previous interventional studies that have demonstrated improvements in biochemical hyperandrogenism, insulin resistance, and menstrual dysfunction following weight loss due to calorie restriction, lifestyle intervention, or bariatric surgey. A study of 24 women with PCOS and obesity, of whom 19 had oligo-amenorrhoea, who underwent caloric restriction to a 1000 kcal per day for 6–7 months demonstrated that in the 13 patients who had more than 5% TWL, there was a significant increase in SHBG (*P* = 0.002), reduction in free testosterone (*P* = 0.009), and reduction in fasting serum insulin concentrations (*P* = 0.018) (Kiddy *et al.*, 1992). Nine of the 11 women with menstrual dysfunction who lost more than 5% of their pretreatment weight had self-reported improvement in menstrual dysfunction and/or spontaneous pregnancy (Kiddy *et al.*, 1992). A more recent prospective study of 33 anovulatory patients with PCOS and overweight prescribed a 1200 kcal per day diet and recommended aerobic exercise, reported resumption of regular menstrual cycles in 72% of patients, with 15 ovulatory cycles during the study period (Crosignani *et al.*, 2003). The study also reported a reduction in ovarian volume by 18% (95% CI 7–29) in women who lost 5%, and by 27% (95% CI 10–41) in those who reached 10% weight loss (Crosignani *et al.*, 2003). A review of observational studies reporting on the effects of bariatric surgery in women with PCOS found that menstrual regularity improved significantly post-operatively (*P* < 0.001) (Samarasinghe *et al.*, 2024b).

We also explored baseline and treatment-related predictors of OvR. Higher baseline AMH concentrations and total testosterone concentrations were associated with a lower likelihood of OvR. As obesity is associated with lower AMH concentrations, it could be expected that weight loss would result in an increase in AMH, however BAMBINI and other studies of weight loss interventions have shown a further reduction in AMH during weight loss (in line with the decrease in ovarian volume mentioned above) (MD −16.8, 95% CI −28.8 to −4.9) (Samarasinghe *et al.*, 2024a). Importantly, as AMH falls from very high (31.3; 95% CI 23.3–39.1) to moderately high concentrations (23.7; 95% CI 15.4–32.0) (Samarasinghe *et al.*, 2024a), this could indicate a degree of normalization of aberrant AMH secretion. Although higher AMH concentrations at baseline were associated with lower odds of recovery of ovulation, the change in AMH after randomization did not predict OvR. This is in keeping with studies demonstrating that high AMH concentration relates to PCOS disease severity (Dewailly *et al.*, 2014), a tempered response to ovulation induction (Mumford *et al.*, 2016), and adverse pregnancy outcomes (Vale-Fernandes *et al.*, 2023). An *in vitro* study in human granulosa cells treated with varying concentrations of AMH found that it inhibits factors that promote follicle progression (Pellatt *et al.*, 2011). A 3-month study of the effects of a hypocaloric dietary intervention in women with PCOS demonstrated that ovarian morphology markers, including follicle number per ovary (*P* = 0.015) and ovarian volume (*P* = 0.001), were decreased post-intervention, but not AMH (*P* = 0.133) (Carter *et al.*, 2025).

Baseline raised total testosterone concentrations were also associated with lower odds of OvR. A hyperandrogenic state significantly inhibits ovarian aromatase activity (independent of BMI) (Chen *et al.*, 2015a), and intraovarian hyperandrogenism is considered an important factor in follicular arrest in both rodent models and women with PCOS (Chen *et al.*, 2015b). While a reduction in total testosterone alone was not predictive, an increase in SHBG and subsequent reduction in free androgen index was associated with a higher likelihood of OvR. This is consistent with experimental and clinical data demonstrating that intraovarian hyperandrogenism disrupts folliculogenesis (Jonard and Dewailly, 2004) and downregulates aromatase activity (Yang *et al.*, 2015).

Analysis of hormone concentrations across 476 reported menstrual cycles identified lower LH, total testosterone, androstenedione, and fasting insulin concentrations during the luteal phase following bariatric surgery compared with medical care. This is in keeping with the known effects of bariatric surgery-related weight loss on these hormones (Eid *et al.*, 2014; Chiofalo *et al.*, 2017; Benito *et al.*, 2020).

In summary, this exploratory *post hoc* analysis supports an association between weight loss and OvR in women with PCOS and obesity (BMI ≥35 kg/m^2^) and suggests that even modest weight loss may improve reproductive function. While OvR was most frequently observed at moderate levels of TWL, these data do not include a large enough sample to define an optimal weight-loss target or confirm that higher degrees of weight loss are without adverse risk. These findings build on the primary BAMBINI clinical trial outcomes by providing mechanistic and reproductive insights and highlighting the need for prospective studies to define clinically relevant weight-loss targets and the long-term reproductive safety of bariatric surgery.

## Data Availability

The data underlying this article will be shared on reasonable request to the corresponding author.
